# Case report: Receptive labeling training in autism: conventional vs. technology-based approaches? a single case study

**DOI:** 10.3389/fpsyt.2024.1437293

**Published:** 2024-12-11

**Authors:** Roberta Minutoli, Ileana Scarcella, Germana Doria, Noemi Vetrano, Paola Chilà, Maria Josè Sireci, Stefania Gismondo, Chiara Failla, Giovanni Pioggia, Flavia Marino

**Affiliations:** ^1^ Institute for Biomedical Research and Innovation (IRIB), National Research Council of Italy (CNR), Messina, Italy; ^2^ Faculty of Psychology, International Telematic University Uninettuno, Roma, Italy; ^3^ Department of Cognitive, Psychological Science and Cultural Studies, University of Messina, Messina, Italy; ^4^ European Institute for the Study of Human Behavior (IESCUM), Parma, Italy; ^5^ Classical Linguistic Studies and Education Department, Kore University of Enna, Enna, Italy

**Keywords:** autism, receptive labeling, conditional-only method, flashcards, tablet

## Abstract

**Background:**

Receptive language, the ability to comprehend and respond to spoken language, poses significant challenges for individuals with Autism Spectrum Disorder (ASD). To support communication in autistic children, interventions like Lovaas’ simple-conditional method and Green’s conditional-only method are commonly employed. Personalized approaches are essential due to the spectrum nature of autism. Advancements in technology have opened new avenues for personalizing therapeutic interventions. This single case study compares traditional and technology-based learning sets in a receptive labeling teaching program using Green’s method.

**Methods:**

An alternating treatments design assessed the number of sessions required to achieve mastery in receptive identification of stimuli presented on flashcards or tablets. The study involved a six-year-old Italian child with ASD named Pietro. Initial assessment using the Verbal Behavior Milestone Assessment and Placement Program (VB-MAPP) determined Pietro’s strengths and weaknesses. Six stimuli were selected and divided into two sets: traditional and technology-based. Sessions were semi-randomly alternated, and the teaching procedures remained constant across conditions. In the traditional condition, sessions were conducted twice a week, using flashcards. Correct responses received immediate social reinforcement. In the technological condition, the same stimuli were presented on a tablet via PowerPoint slides.

**Results:**

Pietro achieved mastery more quickly with flashcard instruction than with tablet instruction. Learning was exponential in the traditional condition and linear in the digital condition. Follow-up assessments three weeks post-treatment showed no differences in the generalization and maintenance of skills between the two modalities.

**Discussion:**

The findings indicate that the format of stimulus delivery affects the learning process, with traditional flashcards leading to faster mastery in this case. Individual motivation appears crucial, suggesting that Pietro’s learning history influenced his performance. Personalized approaches remain vital in autism interventions. Further research is needed to determine if these differences extend to other skills or contexts.

**Conclusion:**

While technology-based interventions offer new opportunities, they are not universally more effective than traditional methods. Careful consideration of individual differences, especially motivational factors, is essential in designing effective autism intervention programs.

## Introduction

In autism, receptive language is the ability to understand and interpret spoken language from others (1). Challenges in this area can lead to difficulties with following instructions, processing information, and engaging in conversations. Acquiring basic receptive language skills is crucial for a child’s overall development and spoken language acquisition, enabling many learning opportunities (2, 3). To support receptive language skills in autistic children, interventions often use Applied Behavior Analysis (ABA) based methods. ABA is the science that studies how the environment influences an individual’s behavior. Through the analysis of these influences, ABA develops interventions aimed at changing behavior. Based on the principles of operant conditioning, ABA aims to assess and reduce dysfunctional behaviors, as well as promote and generalize more adaptive behaviors. Specifically, teaching receptive labeling requires optimized procedures from the earliest stages of programming. With this goal in mind, Grow and LeBlanc (2013) published a set of basic general guidelines that have enabled the planning and implementation of increasingly valid receptive labeling interventions and procedures, as evidenced by subsequent scientific evidence. These include personalized educational interventions wherein healthcare professionals can develop programs tailored to address the specific needs of autistic individuals. The main objective is to act on impaired stimulus regulation, poor attention, and overselection errors - a term used to describe when a child overly focuses on certain stimuli to the exclusion of others, which can significantly hinder their ability to process broader contextual information - that autistic children commonly exhibit in association with the receptive language development (4). Additionally, research has demonstrated that teaching receptive labels has prospective advantages for expressive language, naming, increased engagement, and compliance with rules (5).

The subjectivity of individuals in receptive labeling programs can cause general guidelines to fail, so using alternative strategies (6–9) might be more effective. Nevertheless, two procedures are most commonly utilized in clinical practice for teaching conditional discriminations: the Lovaas’ simple-conditional method (10), which involves progressive introduction of new stimuli, and the Green’s conditional-only method (11), which consist of the simultaneous incorporation of new stimuli (12, 13). The conditional-only method is based on a four-term contingency: (a) a set of comparison stimuli, which include discriminative and distractor stimuli, (b) a corresponding auditory instruction that acts as an antecedent to prompt the behavior, (c) the selection of the appropriate stimulus from the set, which constitutes the targeted behavior, and (d) a reinforcer that follows the behavior, serving to strengthen the likelihood of the correct response in the future. Autism is a spectrum, so receptive language abilities can vary widely among individuals. A personalized approach that takes into account the individual’s needs and abilities is essential to encourage the development of language skills. Over the last decade, advancing technology has played an increasingly significant role in the treatment of autism, providing researchers and clinicians with new opportunities to personalize therapeutic interventions for autistic people (14). Indeed, autistic children may find Information and Communication Technologies (ICT) based interventions applications especially appealing and engaging (15) so computer, tablet, or mobile applications can be promising training tools as long as accompanied by human assistance (16, 17). Most of the articles in the literature on the topic compare the simple-conditional method and the conditional-only method in teaching receptive labeling to autistic children, and most of them demonstrate the greater effectiveness and efficiency of the conditional-only method (13, 18–23). To the best of our knowledge, to date only two studies compared the use of a touch screen device and conventional stimuli in the acquisition of receptive labeling in autistic children (24, 25) but none of them write about the applicability of the conditional-only method with and without the aid of technological tools. The main goal of this single case study is to compare the effectiveness of conventional versus technology-based learning sets in a conditional discrimination program using Green’s method with an autistic child.

## Methods

An alternating treatments design was used to assess the number of sessions needed to achieve mastery criterion in receptive identification of stimuli displayed on flashcards or tablets, while keeping all teaching procedures constant.

### Participants

The research involves a single case study of Pietro, a healthy six-year-old boy from Messina, born at term without complications. He is an only child from a middle socioeconomic background. The parents both enjoy good health with no history of neurological or psychiatric disorders. Pietro, was diagnosed with autism at the age of 28 months which was established by experienced clinicians using the Autism Diagnostic Observation Schedule - Second Edition (ADOS-II) and the Vineland Adaptive Behavior Scales - Second Edition (VABS-II). Pietro has no other relevant co-occurring clinical conditions. The child previously received an intervention related to language skills, specifically Mand (requests that the speaker makes to the listener), Tact (naming/labeling) and Echoic (vocal imitation) verbal operants underlying ABA were taught. The ABA intervention was carried out 2 times a week in a rehabilitation center, achieving the targeted results. During the experimental intervention Pietro did not receive any other type of treatment. During a meeting organized with Pietro and his parents at the Institute for Biomedical Research and Innovation of the National Research Council of Italy (CNR-IRIB) in Messina, the therapists explained the research objectives and collected consent for Pietro to participate in the study. At the initial assessment, the child exhibited mild intellectual disability (Development Quotient, DQ=58.5), language characterized by the production of short sentences (subject+verb), and only occasionally displayed hyperactive behaviors. This classification suggests that Pietro may have difficulties in learning new skills and performing daily activities that are typically expected for his age, necessitating specialized support to enhance his learning and adaptation. The single-case study approach was chosen to closely examine individual learning processes and responses to various teaching methods in children with specific educational needs, enabling precise adjustments to interventions. This method ensures more tailored educational strategies compared to broader group studies. The primary step in the clinical application of ABA is to carefully identify the suitable objectives for each child.

### Measures

**Figure 1:**
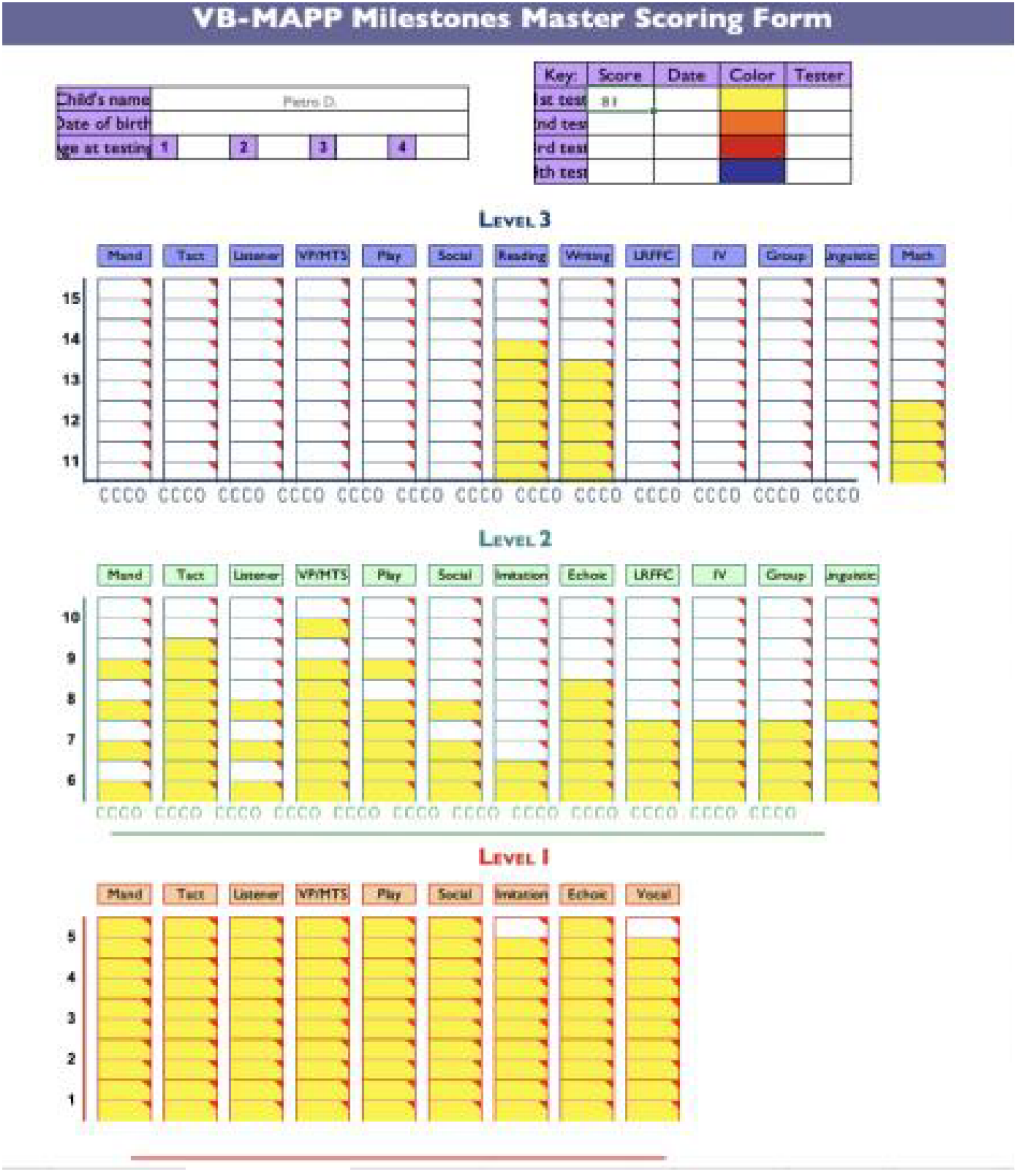
Shows the results of the functional assessment conducted using the Verbal Behavior Milestone Assessment and Placement Program (VB-MAPP).

A comprehensive overview of Pietro’s skills was obtained by using the Verbal Behavior Milestone Assessment and Placement Program (VB-MAPP) (26), a language and social skills assessment program that pays particular attention to all the fundamental competencies for the development, enhancement of verbal communication skills, and the proper use of language in social interactions. Pietro had a VB-MAPP Milestone score of 81, placing him at the 2nd level. The functional analysis highlighted acquired skills in certain areas, including visual-perceptual skills, naming, echoic behavior, reading, and writing. However, there was a need for further development in skills related to requests, imitation, intraverbal behavior, and receptive language, particularly in discriminating against others’ body parts which was identified as a critical area for intervention (see **Figure 1**).

The results indicated that Pietro would benefit from a receptive labeling program. To start, a pre-training assessment was done (baseline) to choose six stimuli, grouped into two sets of three. These sets were then assigned to two teaching methods: traditional and technology-based. During the baseline phase, over three sessions, the instructor showed images of body parts to determine Pietro’s familiarity with them. The instructor assessed the participant’s repertoire by placing three laminated images on the table asking for the target stimulus. The participant had 5 seconds to respond with no further guidance. Target stimuli were then selected based on the percentages of independently correct responses. Specifically, images with high positive response rates (>34%) were considered already learned and established in the child’s learning repertoire, while those with positive response rates ≤33% were included in the receptive labeling teaching program. This approach allowed establishing the child’s prerequisite skills and focusing specifically on the conditional discriminations deemed necessary to develop. Therefore, the target stimuli, identified as calf, nail, wrist, heel, lobe, and palm, were randomly assigned to the two learning sets. The order of conditions varied semi-randomly across sessions.

### Intervention

#### Traditional condition

The nine tasks that constituted each individual treatment session, lasting about 30 minutes each, were conducted twice a week in individual work rooms at the CNR-IRIB in Messina. The total duration of the treatment depended on Pietro’s learning speed. The rooms were equipped with a child-sized table, two chairs, and the necessary materials for conducting the sessions. In the teaching set, the stimuli were aligned in a field of three items to reduce the likelihood of the child responding correctly by chance. In accordance with the four-term contingency that characterizes conditional-only discrimination programs, in each session, first the clinician placed flashcards triplets (printed and laminated images sized 20x25cm) in front of the child. Then, after each trial, the clinician manually rotated each stimulus across either the left, central, and right positions, asking the participant to alternately indicate one of the three body parts assigned to the condition (calf, nail, wrist-image 1/a).

**Figure 2:**
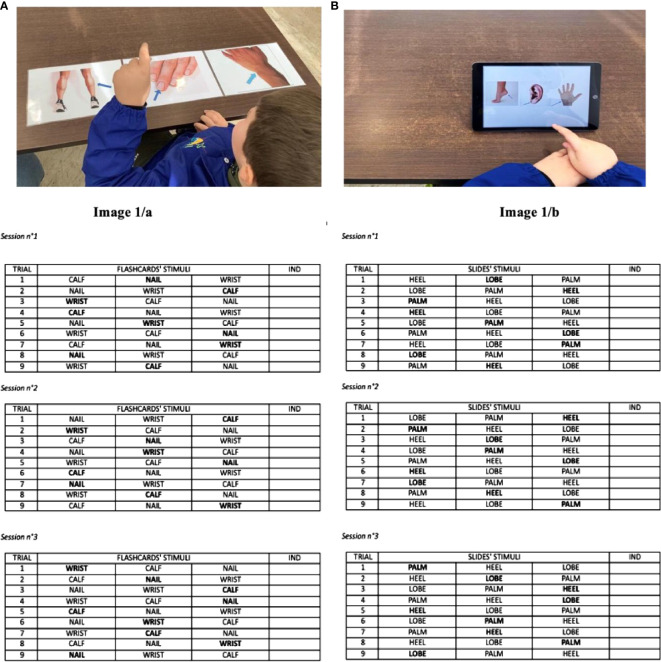
Display the data collection sheet of the receptive language program. The stimuli are presented from the learner's perspective, and the bold stimulus indicates the target (i.e., discriminative stimulus). The data sheet includes three different types of sessions to balance the presentation of stimuli across sessions. **(A)** Data collection sheet of the conventional treatment. **(B)** Data collection sheet of the technological treatment.

As shown in **Figure 2A**, the position and order of stimuli were counterbalanced so that each stimulus was equally targeted across the nine trials per session. Pietro received immediate social reinforcement (“Good job! Well done! You’re great.”) for each correct, independent response. The mastery criterion was achieved with independent identification of the stimulus in at least two consecutive sessions (corresponding to a value of ≥ 89% correct responses across nine trials). A response was deemed correct if the participant identified the visual stimulus within 5 seconds of the auditory cue, without errors or prompts. Three weeks later, a follow-up assessed the generalization and retention of the skills.

#### Technological condition

The teaching procedure was the same as in the traditional condition, except that stimuli were presented digitally. In the experimental condition, stimuli (heel, lobe, palm-image 1/b) appeared on PowerPoint slides on a tablet, each slide showing a different arrangement (see **Figure 2B**). Visual characteristics of the stimuli were standardized for consistency in symbol complexity and color. Data collection, mastery criteria, and follow-up were conducted similarly to the traditional condition.

### Data collection and data analysis

Data were collected during the sessions using specially created pencil-paper grids for the two procedures, following the guidelines outlined by Green (11) to achieve the mastery criterion for receptive labeling. The analysis of the collected data was carried out through a careful visual examination of the graph representing the measured performance. This approach allowed for the identification of possible patterns, trends, and anomalies in the data, facilitating an understanding of the observed phenomena. In particular, the graph was used to highlight the distribution of values, the relationships between variables, and the potential presence of fluctuations or systematic trends over time.

### Validity or reliability

To ensure the validity and reliability of the data collected, rigorous measures were adopted throughout the research process. The team consisted of three main figures: a supervisor, a researcher and a clinician. To ensure the accuracy of the data, scrupulous procedural fidelity checks were carried out. A senior supervisor performed periodic reviews to monitor adherence to the protocol and verify that the intervention was administered consistently and without deviations from the established procedures, observing the sessions from outside through a video camera installed in the room. These checks included careful verification of the correct use of materials, compliance with the times and phases foreseen for each session. During the treatment sessions, the researcher observed the progress of the activities in real time together with the clinician, collecting data with the help of observation grids. The clinician, present in the room with the child, conducted the sessions scrupulously following the guidelines established by the research protocol.

## Results

**Figure 3:**
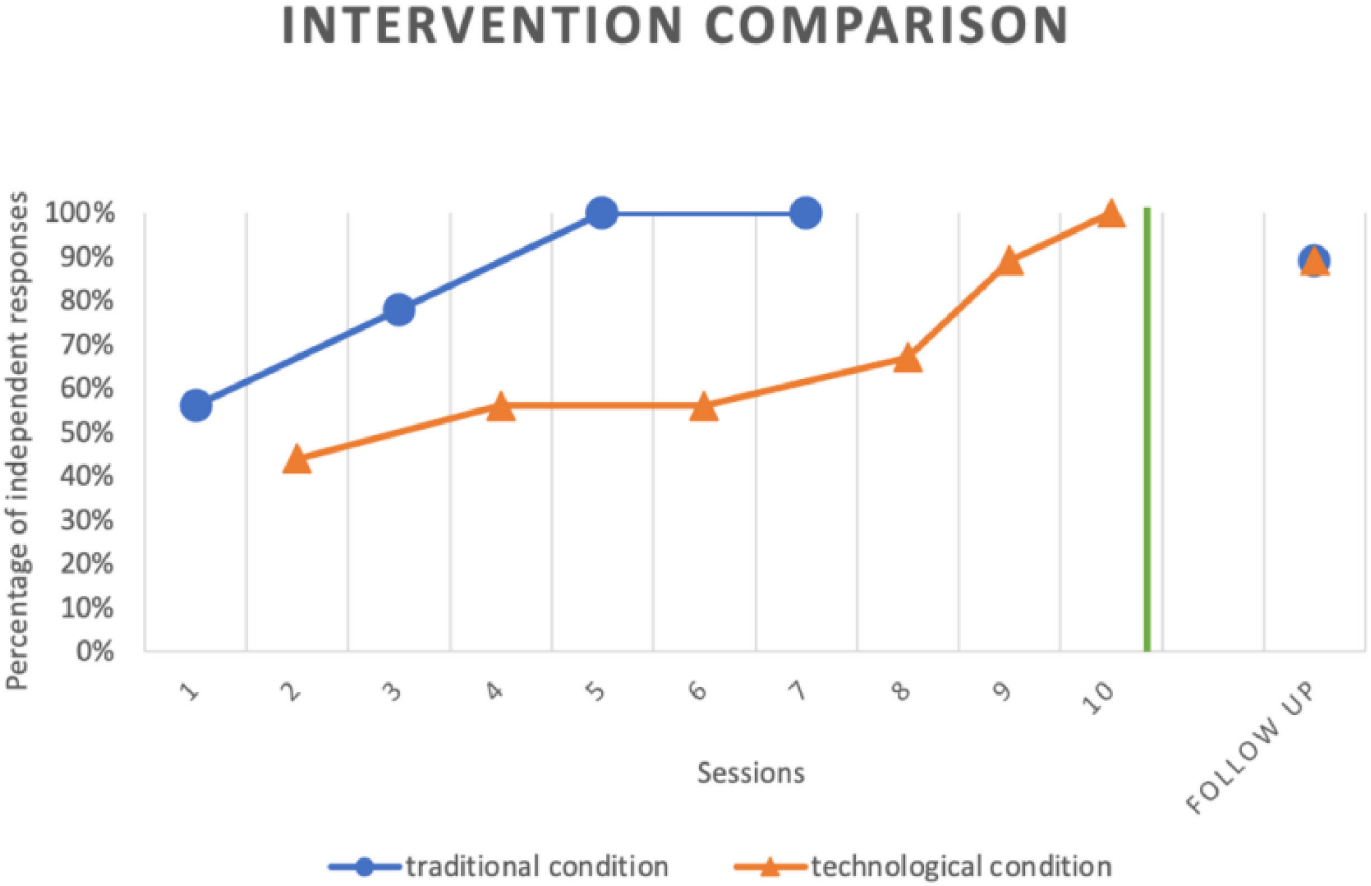
Shows a comparison of the results obtained in both the traditional and technological conditions.

Following the experimental treatment, which spanned a total duration of 5 weeks with bi-weekly sessions, positive outcomes associated with the treatment were achieved. Pietro’s outcomes in the traditional condition reveals the attainment of the mastery criterion in receptive identification of stimuli after just 4 sessions. A more detailed overview of the acquisition trends is presented in **Figure 3**. After the nine trials of the first session, the child achieved a score of 56% in correct and independent responses, accurately labeling five out of the nine stimuli presented in the triplets of flashcards by the experimenter. Subsequent sessions demonstrated a steady improvement in the child’s performance, culminating in the fulfillment of the mastery criterion by the end of the fourth session. In this instance, Pietro once again showcased a percentage of correct responses ≥89%, accurately labeling all the stimuli presented in the nine trials. This rapid attainment of the mastery criterion in the traditional condition underscores the effectiveness of the flashcard-based method for Pietro. The technological condition also yielded positive results, though with a different pattern compared to the traditional condition. The data show that Pietro needed more treatment sessions and a longer time to reach the mastery criterion. Specifically (**Figure 3**), after the first session, Pietro achieved a 44% score in correct and independent responses, responding correctly to four out of nine stimuli within the 5-second limit. In the next two sessions, his score increased slightly to 56% and remained stable before gradually reaching the mastery criterion after 6 sessions and 54 trials in total. Three weeks after treatment, Pietro showed an 89% accuracy rate in both intervention modalities, correctly labeling eight of nine stimuli in both flashcard and tablet conditions. Therefore, Pietro showed good treatment adherence, ensuring that the experimental goal was achieved.

## Discussion

These findings provide insight into how different teaching methods affect receptive labeling skills in autistic individuals. The slight difference in the number of sessions Pietro needed to meet the mastery criterion suggests that the stimulus delivery format can influence learning. The consistency of these findings with a previous study conducted by Pellegrino et al. (24) reinforces the idea of a subtle variation in effectiveness among different stimulus presentation modalities. Interpreting these results requires caution due to the limitations of a single case. Autism is highly individualized, with factors like temperament, personality, environment, and personal motivation playing significant roles. For example, children may react differently to traditional versus technological methods based on their past experiences. Our results suggest that Pietro, having only experienced traditional therapies since about 30 months old, might have been more motivated and therefore more successful with conventional methods. In addition, his young age has led his parents not to expose him to technological tools, such as smartphones or tablets, for long periods of time. This aspect therefore fulfills the proposal of Pellegrino et al. (2019) who suggest studying these procedures (flashcards/technological devices) with participants who have limited history with technological tools. In addition, Pietro showed a clear preference for using flashcards, finding them more stimulating and engaging than the tablet. However, the tablet was requested for playful purposes and not to perform the teaching activity, indicating variability in individual preferences. In line with ABA principles, Pietro’s preference for the traditional method over the technological one underscores the importance of personalized approaches in autism clinical practice, tailored to individual differences. Personalized education plans (IEPs) should incorporate the preferred learning styles and modalities of each child to maximize engagement and effectiveness. Exploring in detail how motivation, exposure to digital devices, and the daily living environment interact with different teaching modalities provides a valuable opportunity to understand what type of treatment may be preferred by the student. Furthermore, it should be noted that, in addition to motivational and methodological aspects, socio-cultural and contextual factors may influence the generalizability of the results. Factors such as the availability of resources, cultural attitudes towards technology, and parental involvement can significantly impact the success of different teaching methods. Bringing attention to these factors is useful to ensure the applicability of conclusions in various contexts and for other autistic individuals. Indeed, the clinical difference observed in Pietro’s case could be attributed to his personal characteristics or be specific to the receptive labeling skills considered in the study. Therefore, exploring whether these differences extend to other competencies or contexts would offer a more comprehensive insight into the effectiveness of interventions. To date, there is a limited number of studies in the literature that promote the teaching of receptive labeling to autistic individuals by comparing the use of flashcards with a technological device. However, none of them strictly applies Green’s (11) conditional-only method. In a study involving a sample of two autistic girls of different ages (3 and 11) Ulzii et al. (25) concluded that tablet-assisted instruction resulted in slightly faster acquisition than flashcards-assisted instruction for both participants. Pellegrino et al. (24), on the other hand, conducted a study in which 2 out of 3 participants required more time to meet a mastery criterion in the tablet condition. Our results contribute to the conclusions drawn by both Pellegrino and Ulzii (24, 25), indicating that both teaching modalities (conventional and technological) promote the acquisition of receptive labeling skills in autistic children, albeit with different timing. This result was also supported by the follow-up conducted three weeks after the treatment, which further suggests that there are no differences in the generalization and maintenance of receptive labeling skills between the two methods. This study demonstrated that the tablet can be used to teach receptive skills, but traditional materials proved to be more efficient. Previous studies by Lee et al. (2015) and Lorah and Karnes (2016) have shown that the tablet can be effective in teaching autistic children if the application is programmed according to research-based interventions (27, 28). Our study adds to existing research by demonstrating that the tablet can indeed be used to teach skills to autistic children using behavioral principles.

### Limitations and future research

The main limitation of this single case study is that the involvement of only one participant limits the generalizability of the findings. Future studies should include a larger and more diverse sample to investigate how these variables interact with different teaching methods. Replicating the procedures with a broader sample could help extend the findings to other populations. Furthermore, despite efforts to control external variables, the researchers cannot fully exclude their potential impact on the results across both teaching conditions. To address this limitation, future research should aim to standardize the stimuli. Looking ahead, further studies are needed to explore how stimuli, teaching methods, and individual traits interact to better address participants’ individual needs and optimize educational outcomes. A multidimensional approach, considering behavioral, cognitive, and environmental factors, is essential for evaluating the effectiveness of interventions and their long-term impact on autistic individuals.

## Conclusion

Technology-based interventions aim to remove barriers impeding autistic individuals from accessing education and communication (14). However, these treatments are not always more effective than traditional ones. The tablet has been in use for a considerable period of time and is considered a valuable and readily accessible tool for implementing intervention programs for individuals with neurodiversity. When utilized effectively, it can expedite learning, reduce human errors in stimulus delivery, minimize inadvertent prompting, and streamline material preparation. These advantages make tablets a compelling option for some learners. Conversely, as widely evidenced, technology-mediated teaching, including tablet-based methods, may potentially hinder skill acquisition due to individual subjectivity. Considering individual differences, particularly motivational factors, is crucial in designing effective intervention programs in autism. IEPs that integrate both traditional and technological methods based on the learner’s strengths and preferences are essential.

## Data Availability

The original contributions presented in the study are included in the article/supplementary material. Further inquiries can be directed to the corresponding author.
